# Searching for a ghost?! The vain ethnobotany of foraging in three coastal Mediterranean areas

**DOI:** 10.1186/s13002-026-00853-0

**Published:** 2026-01-26

**Authors:** Naji Sulaiman, Renata Sõukand, Irfan Ullah, Andrea Pieroni

**Affiliations:** 1 University of Gastronomic Sciences, Piazza Vittorio Emanuele II 9, Pollenzo, 12042 Italy; 2 Department of Environmental Sciences, Informatics and Statistics, Ca’ Foscari University of Venice, Via Torino 155, Venezia, 30172 Italy; 3 Department of Molecular Wood Biotechnology and Technical Mycology, Georg-August University of Göttingen, Büsgenweg 2, 37077 Göttingen, Germany; 4 Department of Medical Analysis, Tishk International University, Erbil, 4001 Iraq

**Keywords:** Ethnobotany, Foraging, Local ecological knowledge, Mediterranean, Land abandonment, Over-Tourism, Desertification

## Abstract

This paper explores the erosion of foraging-related ethnobotanical knowledge in three coastal Mediterranean areas: Gozo Island (Malta), Kasos Island (Greece), and the Castagniccia region of Corsica Island (France). Based on recent ethnobotanical fieldwork between the summer of 2023 and the spring of 2025 in the three study areas, we document the few remaining wild vegetable uses in each region and contextualise the absence of robust LEK linked to plant foraging within broader socio-environmental changes. Our findings show that land abandonment, mass migration, desertification, and the rise of seasonal tourism have contributed to the disintegration of Local Ecological Knowledge (LEK). These forces have severed communities from everyday land-based practices, transforming vibrant ethnobotanical traditions into fragmented memories. We argue that LEK, particularly foraging knowledge, cannot survive in the absence of continuous interaction with the landscape, and that the revival of such practices requires more than nostalgic return, demanding a reconnection with local agro-ecological systems.

## Introduction

 Foraging for wild plants has long been a cornerstone of Mediterranean subsistence culture, intimately linked to food security, medicinal practices, seasonal rhythms, and biocultural identity [1–3]. A growing number of studies have acknowledged that food and ethnobotanical heritage have traditionally played a crucial role in the resilience of local communities, and their potentially significant role in addressing future challenges posed by the turbulent changes affecting food systems worldwide [4, 5]. Across millennia, coastal Mediterranean and Near Eastern landscapes have evolved as complex socio-ecological systems, shaped by traditional agroforestry, grazing, and small-scale polyculture, which not only support biodiversity but also facilitate the intergenerational transmission of place-based knowledge [6–8]. However, the erosion of TEK has become a critical issue as the region faces a phenomenon known as the “Hysteresis Effect”, the idea that, once knowledge systems are lost, they may be difficult or impossible to recover, even when conditions for their re-establishment return [9, 10].

The contemporary Mediterranean, however, is undergoing profound transformations. Since the mid-20th century, rural depopulation, agricultural abandonment, climate pressures, and the rapid growth of mass tourism have all contributed to a disruption in human-environment relationships [11–13]. This has accelerated the erosion of LEK systems, particularly those embedded in everyday practices such as wild plant gathering and seasonal foraging. The socio-ecological impact is evident: collapsed terraces, abandoned groves, and knowledge systems reduced to fragments, with memories held by elders, symbolic gestures, or commodified elements presented to tourists [14]. Once vital practices, such as collecting wild greens or preparing medicinal decoctions, are now marginal in daily life, persisting only as nostalgic traditions [15].

Existing scholarship highlights how abandonment and tourism reshape Mediterranean landscapes in dual and often contradictory ways. On the one hand, ecological succession and reduced human disturbance can facilitate biodiversity recovery; on the other hand, disuse and neglect often degrade terrace infrastructure, provoke erosion, and weaken cultural attachments to the land [16, 17]. Moreover, tourism economies typically favour commodified representations of culture, while devaluing informal knowledge systems and subsistence practices not directly marketable [18]. Studies in regions like Corfu show a measurable decline in the use of wild plants, from 58 foraged species to 42 over just five decades, underscoring the tangible loss of ecological literacy linked to tourism and urbanisation [19].

These processes are particularly evident in marginal coastal Mediterranean regions, peripheral islands, and mountain communities that have historically adapted to low-resource environments. This study focuses on three coastal Mediterranean areas: Gozo (Malta), Kasos Island (Greece), and Castagniccia in Corsica (France). Each of these regions represents a unique biocultural landscape with historically rich foraging traditions, now largely depopulated and increasingly oriented toward tourism or conservation economies. The dynamic systems in these three regions, ranging from chestnut-dominated agroecosystems in Corsica to the dryland gardens of Gozo and the terraced fields of the Aegean, were more than food-producing landscapes: they were repositories of Local Ecological Knowledge (LEK), language, and identity.

Our study, therefore, aims to investigate the extent to which wild vegetable use remains in each of these case study regions and how widely it is retained or practised. It explores how socio-ecological drivers, migration, land abandonment, tourism development, and policy marginalisation contribute to the erosion of LEK and how knowledge may persist in residual or transformed forms. In doing so, we examine whether foraging practices can adapt or regenerate in the face of ecological rupture and cultural change, and what this implies for biocultural resilience in the broader Mediterranean region.

## Methods

### Study areas

**Fig. 1:**
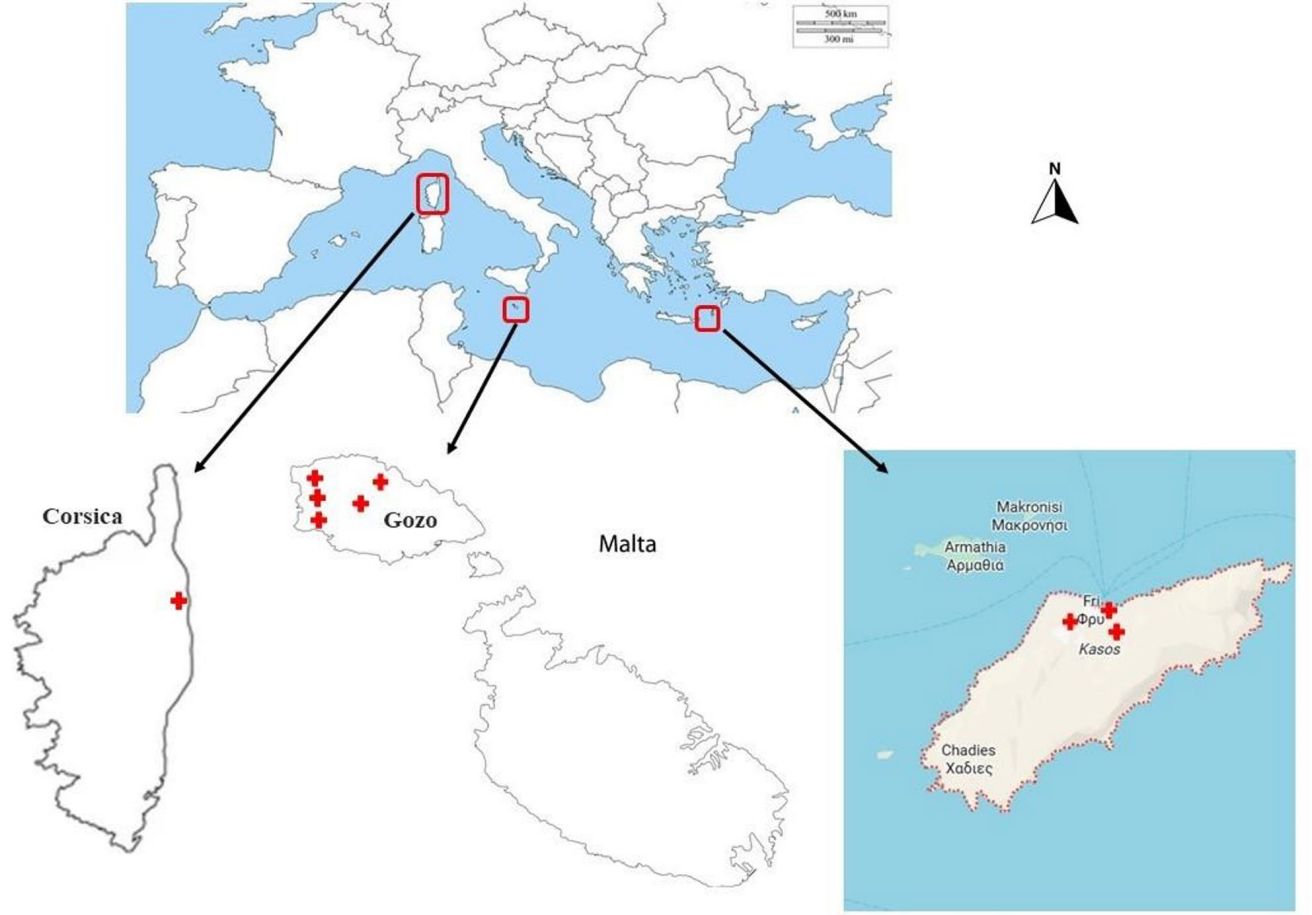
Study area map showing the three study areas. Red crosses refer to the sampled locations

The study was conducted in three Mediterranean areas representing the three Mediterranean geographical regions: the Eastern Mediterranean, with Kasos Island, Greece; the Central Mediterranean, with Gozo Island, Malta; and the Western Mediterranean, with Castagniccia, Corsica, France. Ethnobotanical interviews were conducted in the villages and towns of Marsalforn, Victoria, Gharb, San Lawrenz, and Kercem on the island of Gozo. At the same time, data in Castagniccia, Corsica, were gathered from the villages of Pruno, San Gavino, San Damiano, San Nicolao, La Porta and Cervione. On the other hand, the tiny settlements of Fry, Arvanitochori, and Panagia were sampled in Kasos Island, Greece (Fig. 1).

#### Gozo Island, Malta

**Fig. 2:**
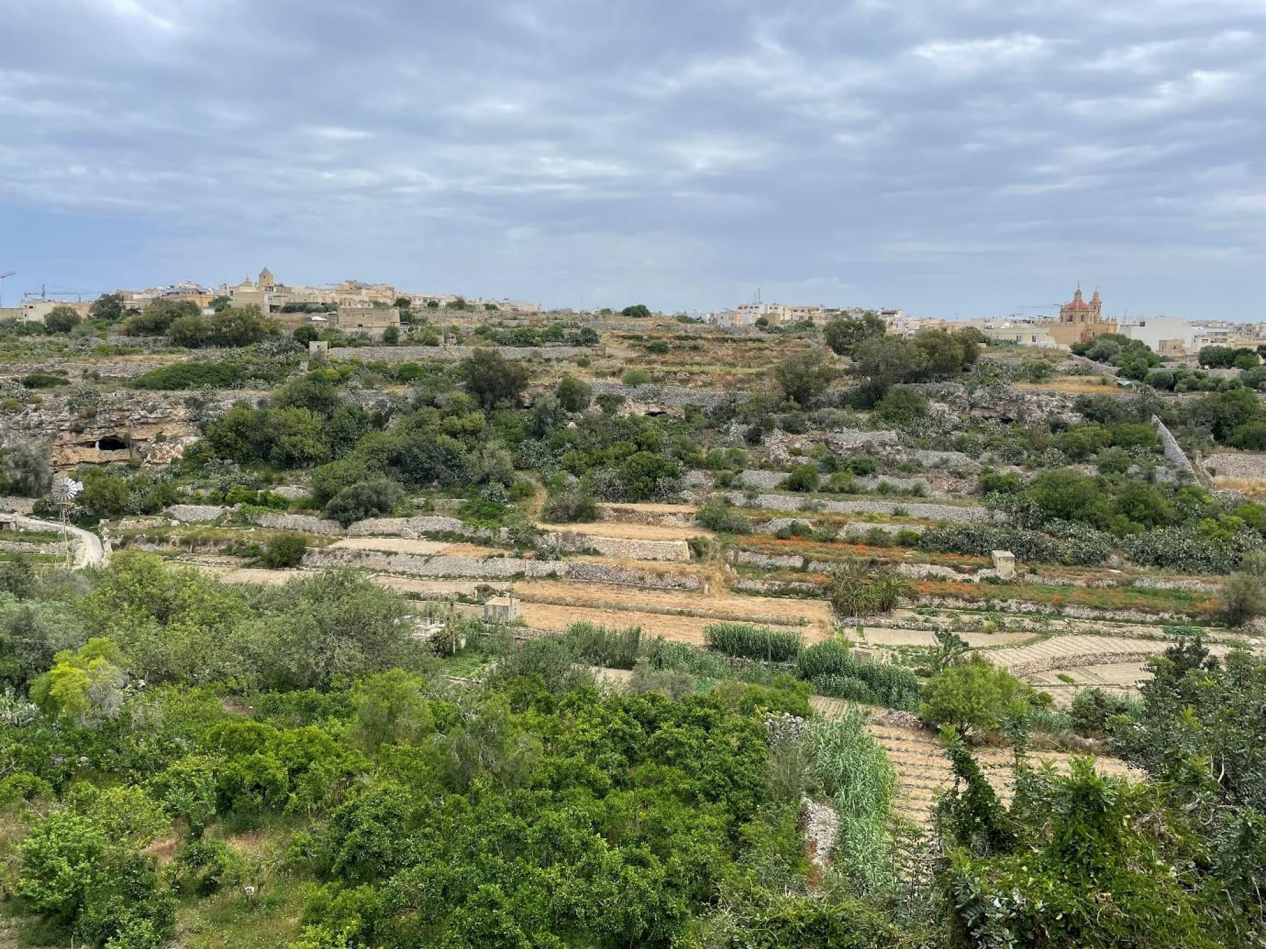
Hilly landscape of the southwestern part of Gozo Island, Malta (Photo credit: N. Sulaiman)

**Fig. 3:**
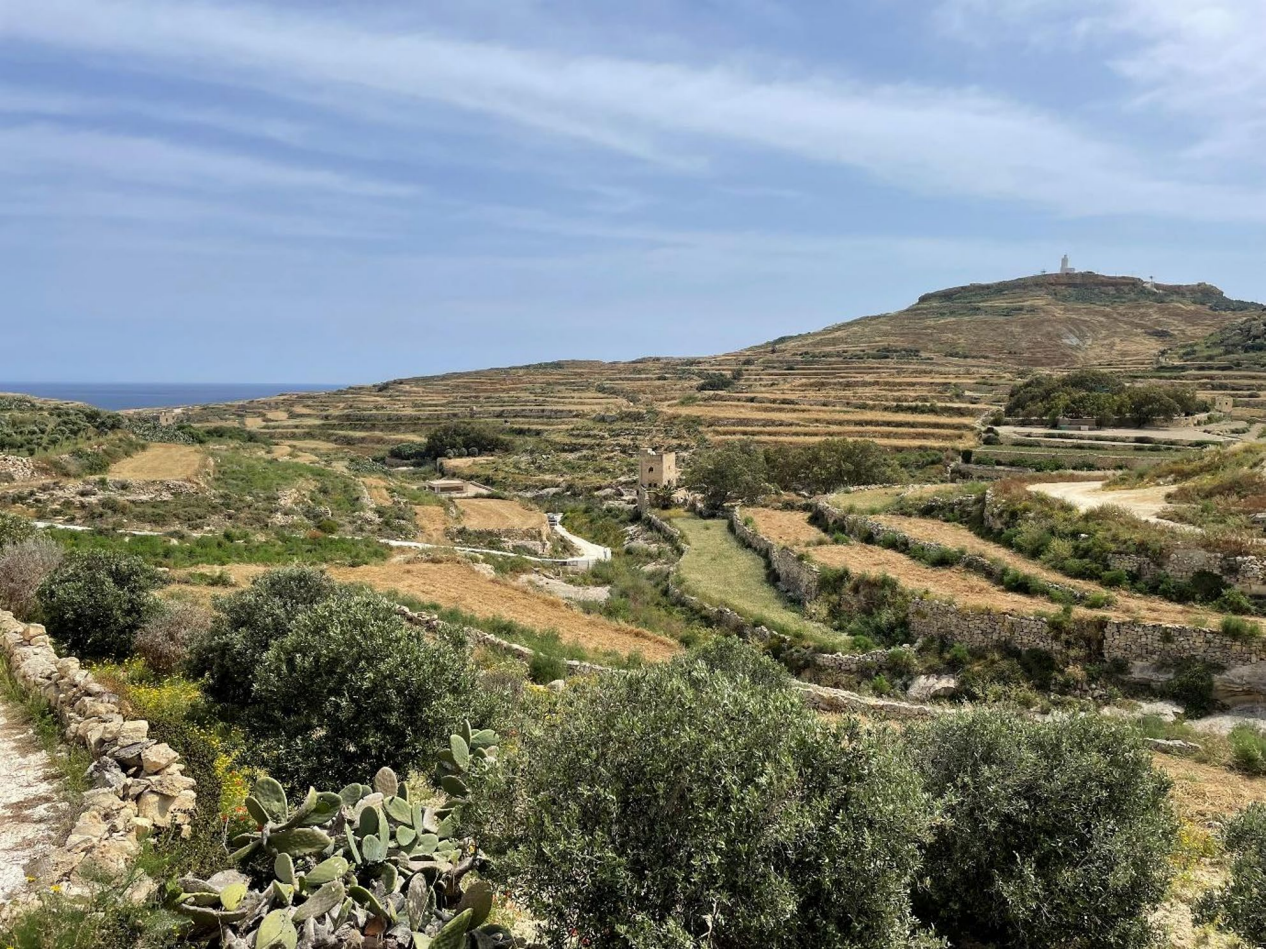
Landscape of Gharb town in Gozo Island, Malta: Olive trees and the Mediterranean Sea in the background. (Photo credit: N. Sulaiman)

Gozo, the second-largest island in the Maltese archipelago, lies in the central Mediterranean Sea and spans an area of approximately 67 square kilometres. Characterised by a rugged coastline, limestone plateaus, and gently undulating hills, the island reaches its highest point at Ta’ Dbiegi (190 m) (Fig. 2). Its landscape is defined by traditional terraced fields built on dry-stone walls, which historically supported diversified subsistence farming. Gozo has a typical Mediterranean climate, with hot, dry summers and mild, wet winters [20]. The island has undergone extensive socio-economic transformations in recent decades. Its population, recorded at around 33,000 in 2021, has become increasingly urbanised, with a significant share engaged in service and tourism sectors [21]. Historically, Gozo’s economy was based on a combination of mixed agriculture, animal husbandry, and maritime trade (Fig. 3). However, following EU accession and a significant increase in tourism, there has been a marked decline in small-scale farming and traditional practices, including the foraging of wild plants [20]. Despite its agrarian heritage, Gozo today is highly dependent on tourism, with rural depopulation and the ageing of farming communities posing threats to the transmission of Local Ecological Knowledge (LEK).

#### Kasos Island, Greece

Kasos Island, located at the southern edge of the Dodecanese in the south-eastern Aegean Sea, is a mountainous island of approximately 49 km². It is among the most remote and least populated islands in Greece, with a 2021 population of fewer than 1,000 residents [22]. Historically a seafaring and agrarian society, Kasos was a thriving maritime hub in the 18th and early 19th centuries, renowned for its trade and shipbuilding. Following destruction during the Greek War of Independence and subsequent waves of emigration, especially in the 20th century, the island suffered dramatic depopulation [23]. Today, many villages are semi-abandoned or occupied only seasonally. The island’s semi-arid climate, combined with overgrazing and rural abandonment, has led to severe land degradation and a shift toward desertification in certain parts of the landscape [24]. Farming practices have largely given way to seasonal tourism. Traditional home gardens and the use of wild plants, once vital to household subsistence, have become vestigial or ritualised memories, especially among older people.

#### Castagniccia, Corsica, France

Castagniccia is a historical region in north-eastern Corsica, France, encompassing a series of mountain villages set within chestnut (*Castanea sativa* Mill.) forests. The region spans approximately 300 square kilometres and is characterised by steep topography and narrow valleys formed over centuries of agro-silvo-pastoral activity. Its climate varies from Mediterranean along the lower slopes to more temperate and humid conditions at higher altitudes. Historically, Castagniccia supported a dense rural population practising diversified small-scale farming, chestnut cultivation, and transhumant livestock herding. During the 19th century, the region was inhabited by over 30,000 people; however, today, fewer than 2,000 permanent residents remain [25]. The collapse of the chestnut economy, combined with emigration and economic centralisation, led to widespread land abandonment. Terraces are overgrown or collapsing, and many villages are only seasonally inhabited, primarily during the summer months. Despite this, Castagniccia retains remnants of biocultural identity, including memory-based plant knowledge linked to chestnut processing, herbal medicine, and abandoned garden plots. The region exemplifies the fragility of LEK in the face of demographic collapse and the challenges of cultural continuity in mountainous landscapes.

### Data collection and analysis

Between July 2023 and May 2025, ethnobotanical data were collected using a qualitative, mixed-methods approach across three Mediterranean regions: Gozo (Malta), Kasos Island (Greece), and Castagniccia (Corsica, France). Fieldwork employed semi-structured interviews with 57 participants (Gozo: 22; Kasos: 16; Castagniccia: 19), primarily elderly residents with long-standing ties to their communities. Participants were primarily recruited using a simple, non-probabilistic snowball sampling approach, commonly employed in qualitative ethnobotanical research to identify individuals with relevant experiential knowledge. Initial interviewees were identified through local contacts and informal conversations in each study area and were selected based on their long-term residence and familiarity with farming, gardening, or land-based practices. Rapport building was pursued where possible during the fieldwork, including informal conversations, shared walks in village streets, gardens and surrounding landscapes [26]. The mean age of respondents was approximately 62 in Gozo, 65 in Kasos, and 60 in Castagniccia. Most participants had engaged in subsistence agriculture, foraging, or pastoralism earlier in their lives.

Interviews were conducted in local dialects or standard languages, depending on participants’ preference, and took place in home gardens, village squares and streets, and occasionally while walking through familiar landscapes. Each interview focused on the past and present use of wild plants, including local vernacular names, plant parts used, preparation methods, seasonal collection habits, and perceived decline or continuity of knowledge. Free listing was employed as a qualitative elicitation technique to explore the presence, absence, and fragmentation of shared ethnobotanical knowledge related to wild plant foraging [27]. In contrast to typical quantitative ethnobotanical studies aimed at ranking culturally salient taxa, this “Analysis” study uses free listing analytically to assess whether a coherent and actively shared body of foraging knowledge persists at all within each study area. Given the very limited number of species cited and the retrospective, narrative character of many interviews, the study was not designed as a salience or ranking analysis. Although frequency information was recorded and is occasionally referenced in the text to support interpretive claims, quantitative indices such as average rank or composite salience were not calculated, as they would not meaningfully serve the aims or context of this manuscript.

To ensure methodological transparency, the research followed the Code of Ethics of the International Society of Ethnobiology [28]. All participants were informed about the study’s goals, and oral informed consent was obtained. The study focused primarily on herbaceous greens and edible plants used in traditional diets and home remedies; however, certain wild fruits that were noticeably emphasised by study participants were also recorded.

Where feasible, voucher specimens were collected and stored in the Herbarium of the University of Gastronomic Sciences and the Herbarium of the Bio-Cultural Diversity Lab at the Department of Environmental Sciences, Informatics, and Statistics, Ca’ Foscari University of Venice, Italy (UVV). For plant taxa that could not be identified in the field, local descriptions were triangulated with photographs, morphological notes, and ecological cues. Photographs of candidate species were also shown to interviewees during a second round of site visits or follow-up sessions. Plant identifications were verified using standard botanical keys and ethnobotanical literature. Plant nomenclature followed The World Flora Online [29]. Local names were transliterated into the Latin alphabet, following established linguistic conventions.

In addition to primary interviews, the study incorporated demographic data from national statistical agencies, including the Hellenic Statistical Authority [22], the National Statistics Office of Malta [21], and the French National Institute of Statistics and Economic Studies [25]. These datasets provided contextual understanding of population changes, urbanisation trends, and rural depopulation relevant to the interpretation of LEK erosion. Quantitative counts (how many respondents cited each species) were recorded. Qualitative analysis focused on themes in the responses, such as memory decline, references to past land-based practices, seasonal cycles, and the social transmission of knowledge. Comparative cross‑regional analysis allowed us to highlight both shared forces of decline and region‑specific trajectories.

## Results and discussion

### Gozo, Malta

Study participants in Gozo recalled a modest selection of wild food plants, with only three taxa, *Opuntia ficus-indica* (L.) Mill., *Laurus nobilis* L., and *Urtica dioica* L., were mentioned repeatedly across interviews, indicating very limited shared knowledge and low intersubjective consensus. Table 1 summarises the eight taxa identified, including botanical and folk names, plant parts used, traditional culinary uses, and corresponding voucher specimens.

Despite the island’s historically terraced agroecosystems and proximity to biodiverse scrublands, the current foraging repertoire appears limited. This is especially notable given Gozo’s past reliance on wild plants during periods of resource scarcity, particularly before EU accession and increased food imports. The plants remembered or still used include *Capparis spinosa* L., once harvested for its pickled flower buds, and *Malva sylvestris* L., traditionally infused for digestive ailments or incorporated into pies. *Ceratonia siliqua* L., or carob, was recalled by a few older informants as part of “Gulab,” a sweet preparation with nostalgic associations but no current use.

**Fig. 4:**
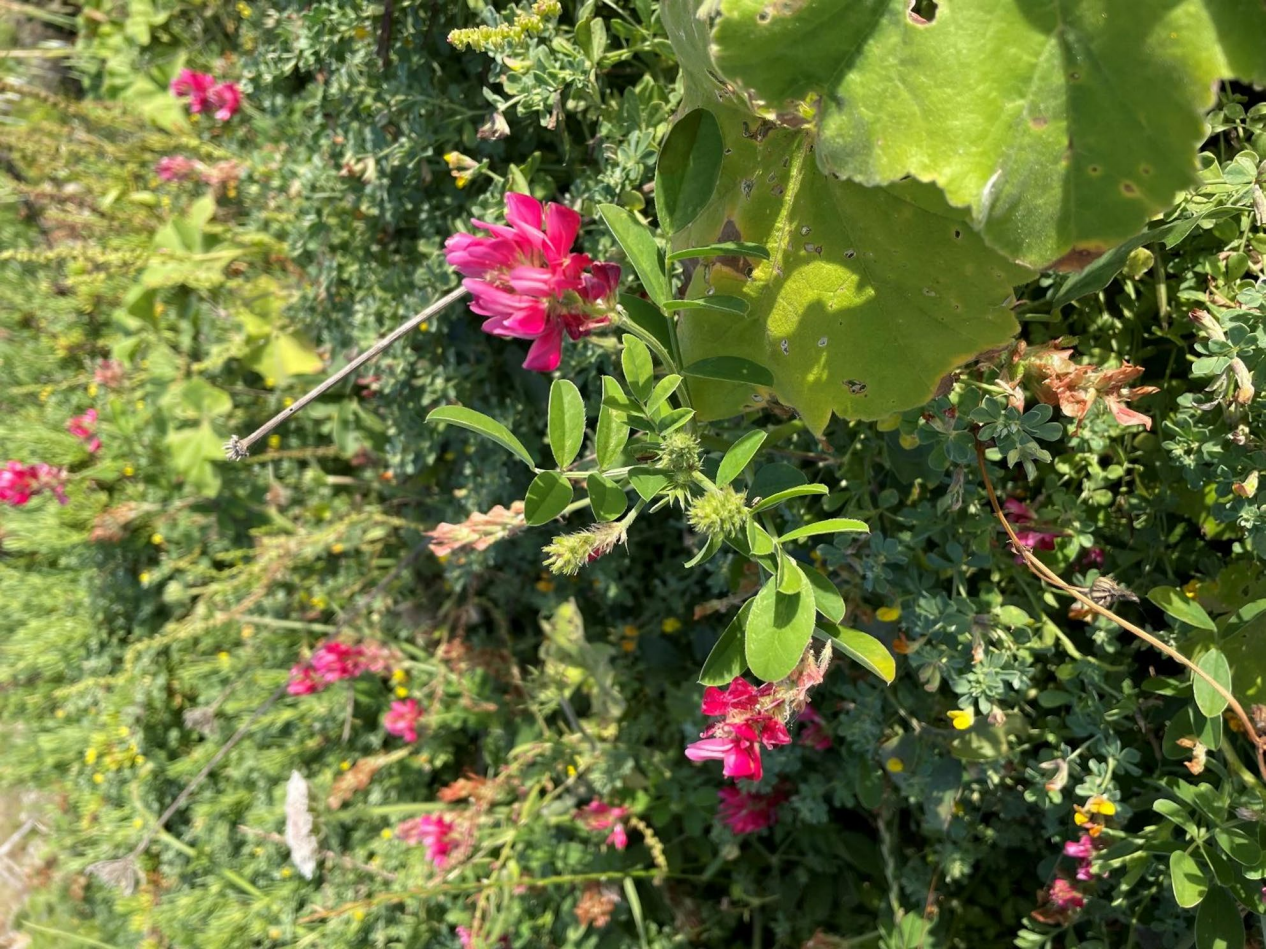
*Sulla coronaria* in wild areas of Gozo, Malta. (Photo credit: N. Sulaiman)

The fruit of *Opuntia* remains one of the few wild plants commonly consumed, often gathered in late summer and eaten fresh or made into jams. *Thymus capitatus* (L.) Cav. (locally Saghtar) was mentioned as a seasoning for meats, reflecting its enduring aromatic role. However, species such as *Sulla coronaria* (L.) B.H.Choi & H.Ohashi (Fig. 4) and *Capparis spinosa*, while still growing abundantly on rocky outcrops and terraces, are no longer foraged by younger generations. Accounts of earlier foraging practices are based on first-person life-history narratives of elderly participants, who described gathering these species during childhood or early adulthood as part of everyday household food practices. In contrast, younger informants neither reported recognising nor using these plants, indicating a disruption in intergenerational transmission rather than an absence of historical use. This loss of plant uses points to a rupture in both ecological knowledge and culinary transmission.

These findings align with broader trends observed in Malta and other island territories, where increased reliance on industrially produced foods, the decline of intergenerational farming, and the loss of rural labour economies have contributed to the marginalisation of wild food traditions. In Gozo, tourism and infrastructure development have further severed the link between landscape and livelihood, with many former agricultural areas now dominated by absentee ownership or seasonal occupancy. The erosion of LEK observed here mirrors patterns in other Mediterranean contexts where socio-ecological transformations diminish community reliance on wild flora [11].

**Table 1:** Wild food plants reported by study participants in Gozo, Malta

Botanical name; voucher specimens	Family	Local name	Parts used	Use
*Capparis spinosa* L.	Capparaceae	Caper	Flower buds	Pickled
*Ceratonia siliqua* L. UNISGNS2025001	Fabaceae	Harruba	Pods and seeds	Traditional Gulab preparation
*Sulla coronaria* (L.) B.H.Choi & H.Ohashi UNISGNS2025002	Fabaceae	Silla	Leaves, pods	Eaten fresh
*Laurus nobilis* L.	Lauraceae	Randa	Leaves	Flavouring soups and sauces
*Malva sylvestris* L. UNISGNS2025003	Malvaceae	Hubbejża	Leaves, flowers	Herbal tea, pie filling
*Opuntia ficus-indica* (L.) Mill	Cactaceae	Bajtar tax-xewk	Fruit	Eaten fresh, preserved
*Thymus capitatus* (L.) Cav.	Lamiaceae	Saghtar	Young aerial part	Flavouring meats, soups
*Urtica dioica* L. UNISGNS2025004	Urticaceae	Hurrieq	Leaves	Leaves are used in soups, stews, and as a salad ingredient

The relatively low diversity and consensus of cited taxa underscore the accelerated pace of cultural and ecological disconnection. Informants frequently contextualised their knowledge in the past tense, referring to the plants as “things we used to pick” or “foods from before the shops came”. While a few participants still gathered *Opuntia* or dried leaves of *Laurus nobilis* L. for cooking, most plant uses were remembered rather than practised, reinforcing the idea of LEK survival through memory and nostalgia rather than daily life.

### Kasos Island, Greece

Respondents identified nine wild edible plants traditionally gathered and consumed on the island (Table 2), each referred to by local Greek names and associated with distinct seasonal and culinary practices.

Kasos, a remote and semi-arid island in the southern Dodecanese, has experienced significant socio-ecological shifts over the past half-century. The abandonment of terraced agriculture, widespread depopulation, and increasing desertification have contributed to the erosion of traditional ecological knowledge (TEK). Although plant diversity in foraging was once robust, reflecting a diet adapted to a harsh, low-resource environment, our findings reveal that this legacy is now primarily confined to memory. The wild greens cited most frequently included *Capparis spinosa* L. (pickled), *Cichorium spinosissimum* Jacq. (salads or boiled), and *Sonchus* spp., which are commonly consumed when cooked in mixed dishes. However, only a minority of interviewees still gather these plants actively.

Table 2 lists the plant taxa reported, including botanical and local names, parts used, and traditional culinary applications. Notably, many of the species are bitter greens from the Asteraceae family, a characteristic pattern observed in other Dodecanese and Cycladic islands [30]. These plants were once integral to the local diet, either gathered in winter and early spring or preserved through drying and pickling. *Crithmum maritimum* L., growing along rocky shorelines, was often pickled and stored in vinegar, while *Foeniculum vulgare* Mill., and *Origanum vulgare* subsp. *hirtum* (Link) A.Terracc. continues to be used sparingly for flavouring.

Despite the observed diversity, the intergenerational transmission of knowledge appears critically endangered. Most informants emphasised that younger residents “*don’t even know these names*”, with one participant stating: “*Now it’s all ended*,* dead*”, a powerful expression of the emotional and cultural rupture caused by rural abandonment. This resonates with broader findings across the Aegean, where LEK is rapidly vanishing due to demographic ageing, migration, and a loss of subsistence-oriented lifestyles [31].

Kasos has suffered from one of the highest rates of depopulation in the Dodecanese, and its local economy, once rooted in pastoralism, fishing, and self-sufficient agriculture, has shifted toward seasonal, diaspora-driven tourism. Agricultural terraces are now largely abandoned, and home gardens, formerly maintained even during drought years, are rare. The collapse of these local systems has directly undermined the context in which plant knowledge was practised, leading to what Berkes et al. [32] term the “erosion of the knowledge-practice-belief complex”.

**Table 2:** Wild food plants reported by study participants in Kasos, Greece

Botanical name	Family	Local name	Parts used	Use
*Capparis spinosa subsp. rupestris* (Sm.) Nyman	Capparaceae	Kapari	Buds/fruits	Pickled
*Cichorium spinosissimum* Jacq.	Asteraceae	Roikio	Whorls	Salads, cooked
*Crithmum maritimum* L.	Apiaceae	Kritama	Young shoots	Pickled
*Foeniculum vulgare* Mill.	Apiaceae	Marathos	Shoots/fruits	Cooked/seasoning
*Glebionis coronaria* (L.) Cass. ex Spach	Asteraceae	Amarangos	Leaves	Salad
*Malva sylvestris* L.	Malvaceae	Amolochas	Leaves	Cooked
*Origanum vulgare subsp. hirtum* (Link) A.Terracc.	Lamiaceae	Rigani	Flowering tops	Seasoning
*Scolymus hispanicus* L.	Asteraceae	Akanos	Young stems	Cooked
*Sonchus* spp.	Asteraceae	Zochos	Whorls	Cooked

The disappearance of both practice and knowledge is symptomatic of a broader socio-ecological transformation in Kasos. While the biodiversity remains relatively intact, the absence of active use signifies what Turner and Turner [33] have termed “cultural extinction”, the loss of knowledge before the loss of species. In this context, conservation efforts must consider not only plant habitats but also the cultural frameworks through which they are known and used.

### Castagniccia, Corsica

Ethnobotanical data collected from this area revealed only two wild taxa actively cited as used for food: *Clinopodium nepeta* (L.) Kuntze and *Asparagus acutifolius* L. (Table 3). This is in sharp contrast to an ethnobotanical inventory from the early 1980 s, which documented more than two dozen wild edible greens traditionally gathered in the region [34].

**Table 3:** Wild food plants reported by study participants in Castagniccia, Corsica

Botanical name; voucher specimens	Family	Local name	Parts used	Use
*Clinopodium nepeta* (L.) Kuntze, UVVCOR01	Lamiaceae	Nepita	Aerial parts	Infusion, seasoning for meats and stews
*Asparagus acutifolius* L. UVVCOR02	Asparagaceae	Sparaghi selvatici	Young shoots	Cooked with eggs or in omelettes

The historical dataset included a range of commonly foraged species such as *Silene vulgaris* (Moench) Garcke, *Borago officinalis* L., *Rumex* spp., *Papaver rhoeas* L., and *Allium ampeloprasum* L., many of which are absent from recent narratives. The disappearance of such a rich assemblage signals a profound collapse in local ecological knowledge (LEK) tied to foraging and seasonal plant use. The decline is not only quantitative but also qualitative, affecting the diversity of preparation methods, sensory vocabulary, and seasonal practices that once characterised Corsican foodways.

The two species still cited, *Clinopodium nepeta* (used as an herbal infusion or seasoning) and *Asparagus acutifolius* (young shoots gathered in early spring and consumed boiled or in omelettes), are also known outside Corsica and continue to be used sporadically in modern kitchens, often more as nostalgic symbols than subsistence necessities. Notably, both are perennial and resilient, and their habitats remain relatively undisturbed in forest edges and abandoned field margins, unlike more ephemeral taxa that require human-managed ecotones [35].

**Fig. 5:**
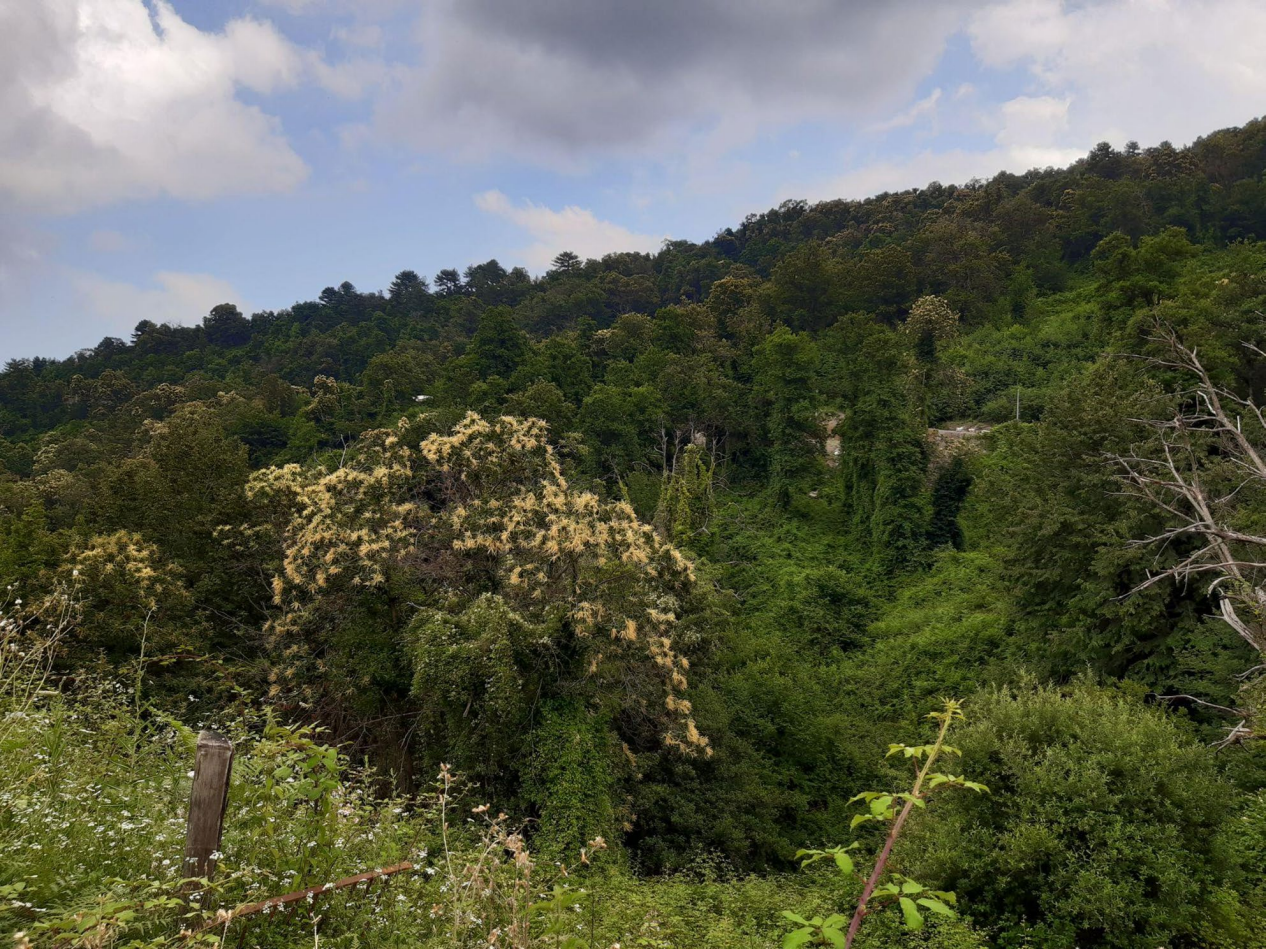
Abandoned gardens taken over by wilderness. La Porta, Castagniccia. (Photo credit: R. Sõukand)

This knowledge loss is closely tied to broader structural transformations. The Castagniccia region has experienced intense depopulation since the mid-20th century, with many residents migrating to cities or mainland France. Once densely cultivated terraces and chestnut groves, formerly the backbone of Corsican agro-silvopastoralism, now lie abandoned (Fig. 5), and the intricate water management systems (fountains, canals, aghje) have fallen into disrepair.

Seasonal gentrification has exacerbated this disconnection. Many of the current homeowners are affluent Corsican émigrés or metropolitan French citizens who occupy homes during summer months but do not engage in land-based livelihoods. This creates a cultural and ecological vacuum wherein LEK is no longer embedded in daily life or landscape management. As Berkes [6] notes, when knowledge becomes “decoupled” from practice and stewardship, it tends to erode rapidly, even in ecologically intact settings.

Thus, while plant biodiversity remains relatively high in Castagniccia, the embedded cultural mechanisms for transmission, renewal, and application of knowledge have largely dissolved. This case exemplifies the “empty landscape paradox,” where heritage-rich ecologies persist in form but not in cultural function [36].

The striking disparity between past and present ethnobotanical repertoires in Castagniccia highlights the fragility of LEK systems amid socio-demographic and land use changes. Preservation efforts must move beyond simple documentation and instead explore re-linking knowledge to land stewardship and community identity [37].

### The unravelling of knowledge landscapes: from practice to nostalgia

For Gozo and Kasos, the absence of prior ethnobotanical studies precludes direct diachronic comparison. In these cases, interpretations of ethnobotanical erosion are based on elderly participants’ life-history narratives, socio-demographic transformations, and comparison with ethnobotanical records from ecologically and culturally comparable Mediterranean regions characterised by shared floristic assemblages and foraging traditions [12, 15, 19, 35].

Across the Mediterranean sites of Gozo (Malta), Kasos (Greece), and Castagniccia (Corsica), a consistent and alarming pattern emerges: the decline of Local Ecological Knowledge (LEK) is closely tied to the erosion of everyday land-based practices and community demographics. Traditional ecological knowledge is not a static inventory of species or techniques, but a dynamic, place-based system of understanding, deeply embedded in daily interactions with the land, structured through rhythms of labour, seasonality, and intergenerational transmission [6, 11, 38].

This study is informed by perspectives from political ecology and political ethnobiology, which emphasise that the erosion of Local Ecological Knowledge cannot be understood independently of broader power relations, governance frameworks, and land-use transformations [39, 40]. From this perspective, the contraction of foraging-related knowledge observed in the study areas is interpreted as a structurally mediated process linked to uneven socio-economic reorganisation, rather than as an isolated or purely cultural phenomenon.

Across the three sites, the agents driving LEK erosion are not abstract forces but identifiable actors. Tourism development is largely fuelled by a mix of domestic capital and small-scale external investment, often incentivised by national and EU-level development frameworks. Local governments, through zoning, infrastructure expansion, and heritage branding, frequently privilege short-term economic growth over land-based livelihoods. While multilateral bodies such as UNESCO promote heritage safeguarding, their influence remains largely symbolic unless coupled with policies that sustain everyday practice rather than curated representation.

What becomes apparent in all three cases is that when land use ceases, LEK ceases to evolve and adapt; it lingers only in memory, if at all. In Castagniccia, once home to intricate chestnut agroforestry systems and managed terraces, interview data show that only two wild edible species are currently remembered and used, down from fifteen reported in the 1980 s [34]. This collapse in ethnobotanical diversity mirrors the physical collapse of the terraces and the water infrastructure, which once maintained a mosaic of habitats and food systems. Ecological degradation, in this context, is not only environmental; it is cognitive and cultural.

The resurgence of summer presence by returnees or affluent French holiday homeowners in Corsica adds a complex layer to this loss. These seasonal residents invest in social and aesthetic revival, festivals, and home restorations, but not in productive and continuous engagement with the local landscape. The landscape becomes a backdrop for memory and consumption rather than a space of ongoing stewardship. As a result, LEK becomes decoupled from land: people may remember the name of a plant or taste its pickled version at a feast, but they no longer know how to find it, recognise it, or harvest it sustainably [41]. The disembodying of knowledge from practice converts LEK from a living tradition into cultural residue [6, 42].

Kasos tells a similar story, with even more severe ecological consequences. A historically biodiverse island, Kasos has undergone drastic desertification following the mid-20th-century collapse of local agriculture and mass emigration. Home gardens and smallholdings, once the keystone of both subsistence and LEK transmission, have almost entirely vanished. Only a handful of elderly locals now recall and utilise wild edible plants, and even among them, the number of species used is minimal. The physical degradation of terraces and fields, compounded by invasive shrub encroachment and soil erosion, renders restoration of former agroecological regimes increasingly unlikely. When informants lament “*now it’s all ended*,” they are not merely describing a lost practice but mourning an entire way of knowing and inhabiting the land. 

Gozo, although demographically more stable, reveals a subtler erosion: LEK persists primarily as a culinary memory, not as a living ecological engagement, as an element representing unlearning debt, where the memory is still present, yet the practice is not carried on nor transmitted [43]. While some species, such as *Opuntia* or *Laurus nobilis*, are still used, their consumption is largely symbolic or nostalgic rather than integral to subsistence cycles. The influx of imported food and market dependence has displaced both the necessity and the know-how of wild foraging. LEK, when disconnected from practical application, becomes vulnerable to selective retention, often favouring plants with culinary prestige or aesthetic value over those critical for ecological management or everyday use.

In sum, these cases illustrate that LEK is not simply lost through forgetting, but through disuse, through the systematic erosion of the conditions that once made it necessary and learnable. Land abandonment, demographic thinning, and the monetisation of place through tourism form a web of drivers that not only interrupt knowledge transmission but also dissolve the very landscapes of memory and labour where such knowledge once flourished.

### The structural disembodying of LEK: socioeconomic transformation and the tourism trap

The regional data also point to a more profound structural crisis: LEK is being disembodied not merely through individual forgetfulness or ageing, but through the reorganisation of rural Mediterranean economies and societies. Specifically, the shift toward tourism-based development, coupled with broader processes of globalisation and market integration, has fundamentally reshaped the ecological and cultural functions of rural landscapes [14, 44].

In Kasos and Castagniccia, tourism manifests as seasonal visitation, often by return migrants or foreign second-home owners. These visitors contribute socially and economically, but their engagement with the land is superficial or nostalgic. They do not maintain terraces, manage irrigation systems, or cultivate home gardens; instead, they consume local products as cultural signifiers. This is what Barthel et al. [42] term “shallow memory practices”, activities that evoke traditional lifeways but lack the practical and ecological feedback loops necessary to regenerate knowledge. The result is a performance of heritage, disconnected from the systems that once gave it life.

In Gozo, the commodification of food and the island’s increasing integration into global supply chains have led to a shift from subsistence-oriented plant use to market dependence. Informants speak fondly of dishes that once relied on wild edibles, but few still forage or pass on the knowledge. When food is bought and consumed in disconnected settings (supermarkets, restaurants), the embodied, intergenerational understanding of where, when, and how to find wild food is no longer transmitted.

This reflects a broader Mediterranean trend: LEK is incompatible with dislocated consumption. It requires rootedness, proximity, repetition, and experiential learning. Once tourism economies dominate, landscapes are transformed not into sites of work and education, but into spaces of leisure and spectacle. This logic of extraction, where the value of a place lies in its marketability, not its liveability, contributes to both ecological and epistemic erosion. Rural space becomes what sociologist Henri Lefebvre called “abstract space”, designed for circulation and profit, not for situated, relational practice [45].

Furthermore, LEK cannot be preserved solely through documentation. While recording species, uses, and vernacular names is crucial for biocultural heritage, codified knowledge divorced from use risks becoming archival. As Berkes [6] insists, LEK is not reducible to lists; it is a practice ecology, one that demands ongoing engagement with soils, climates, plants, and animals. Reviving it requires not just memory or education, but also the material conditions, time, space, land access, and intergenerational households that make such learning viable. This will help to carry on and help to evolve the tacit, inexpressible knowledge that can only be transmitted through experience.

Therefore, any serious attempt to conserve LEK must go beyond symbolic gestures or cultural programming. It must involve strategies that re-couple people and place: land access programs for young farmers, restoration of communal infrastructures (terraces, cisterns, pastures), and incentives for small-scale, biodiversity-oriented cultivation. As Alrhmoun et al. [19] argue in their study of Corfu, without deliberate efforts to re-anchor people in ecological practice, Mediterranean LEK systems are unlikely to survive more than another generation.

The erosion of foraging knowledge also intersects with broader dietary transitions documented across Mediterranean societies, where the replacement of seasonal, plant-rich diets with processed foods has been associated with rising rates of metabolic disorders [46, 47]. While our study does not assess health outcomes directly, the disappearance of wild greens from everyday diets represents not only a cultural loss but a potential nutritional shift with long-term implications.

## Conclusion

This study highlights a profound and accelerating decline in Local Ecological Knowledge (LEK) related to wild plant foraging across three Mediterranean contexts: Gozo, Kasos, and Castagniccia. The sparse and fragmented ethnobotanical citations recorded do not merely represent loss of botanical names or uses but signal the broader disintegration of cultural landscapes and community stewardship practices that sustained these knowledge systems for generations. Our findings reveal how demographic collapse, land abandonment, ecological degradation, and the rise of seasonal, tourism-driven economies form a tightly interconnected web that disrupts the intergenerational transmission and active practice of LEK.

Importantly, LEK cannot be reclaimed through episodic or symbolic gestures alone, such as cultural festivals, heritage branding, or biocultural tourism, because these approaches often emphasise memory over practice. Our data demonstrate that nostalgia and intermittent seasonal presence may evoke emotional connection but do not substitute for the sustained, lived engagement with multifunctional agroecosystems that nurture knowledge renewal, adaptation, and resilience [6, 42]. Instead, the revival of Mediterranean ethnobotanical heritage must be grounded in year-round, place-based ecological interactions: active stewardship of terraces and water infrastructure, restoration of home gardens, and the facilitation of intergenerational apprenticeship where elders and youth collaboratively engage in wild plant cultivation, sustainable foraging, and landscape care.

Such efforts require an integrated socio-ecological approach that acknowledges LEK as a practice ecology, a relational, dynamic system dependent on embeddedness within the lived environment and community livelihoods [11], with its tacit and unacknowledged constituents [41]. Without this, wild plant knowledge risks becoming a spectral vestige: present only as fragmented culinary lore or cultural memory, increasingly detached from real-world ecological conditions and deprived of its functional role in food security, biodiversity maintenance, and cultural identity.

Therefore, effective intervention must transcend tourism commodification and episodic conservation. It calls for policy frameworks and community initiatives that promote land access for small-scale farmers, support the restoration of agroecological infrastructures, and integrate LEK into formal and informal education systems. Only through the re-coupling of people, place, and practice can these Mediterranean landscapes regain their biocultural vitality, ensuring that local ecological knowledge is not lost but revitalised as a living, adaptive heritage for future generations.

The rapid decline of Local Ecological Knowledge (LEK) in Mediterranean contexts, particularly related to foraging and ethnobotanical practices, is primarily attributed to significant socio-environmental changes. This erosion has been intensified by widespread land abandonment, mass migration, and the increasing dominance of seasonal tourism within these coastal regions. Such shifts have severed the connection between local communities and their landscapes, leading to a documented loss of traditional foraging practices that were once integral to regional identity and sustenance [48–50].

Empirical evidence highlights that as communities disengage from everyday interactions with nature, the dynamic systems that sustain LEK become fragmented, transforming vibrant cultural practices into mere vestiges of memory [51, 52]. For example, in regions like Gozo and Castagniccia, traditional knowledge related to wild vegetable use has sharply diminished, reflective of broader patterns in Mediterranean socio-ecological contexts that prioritise commodification over sustainability [53, 54]. This trend signifies not only a loss of practical knowledge but also an underlying cultural shift that impacts social cohesion and community resilience [52, 55].

To revitalise LEK within these Mediterranean areas, it is essential to reintegrate local communities into their agro-ecological systems actively. Revitalisation efforts must focus on participatory decision-making processes that empower community members to reclaim their roles as custodians of traditional knowledge [54]. Engaging stakeholders in conservation initiatives, supported by education programs around sustainable practices, can catalyse both the preservation of LEK and the enhancement of ecological health [52, 54, 55]. Furthermore, leveraging innovations such as community-based resource management can facilitate the documentation and sharing of LEK in a manner that values and respects indigenous perspectives while adapting to contemporary challenges [56, 57].

Ultimately, the maintenance and rejuvenation of LEK in the Mediterranean context depend upon a collaborative approach that interweaves modern scientific understanding with traditional ecological insights, fostering both conservation and cultural identity among local populations [58]. By addressing the socio-political and environmental factors contributing to LEK decline and promoting active community engagement, it is possible to chart a path toward a sustainable and culturally rich future for Mediterranean communities.

## Data Availability

The data supporting this study’s findings are presented in this article.
